# Daily quality assurance, system and procedure‐specific commissioning of a bore‐based optical surface imaging system for surface‐guided radiotherapy (SGRT)

**DOI:** 10.1002/acm2.70586

**Published:** 2026-04-15

**Authors:** Guang Li, Xiaoning Liu, William Donahue, Shih‐Chi Lin, Chengzhu Zhang, Wendy Harris, David Kanchaveli, Seng Boh Lim, Michell Savacool, Michalis Aristophanous, Laura Cervino

**Affiliations:** ^1^ Department of Medical Physics Memorial Sloan Kettering Cancer Center New York City New York USA

**Keywords:** calibration, daily collision check for bore‐based Linacs, optical surface imaging (OSI), QA and commissioning, surface‐guided radiotherapy (SGRT)

## Abstract

**Purpose:**

This study aimed to commission the AlignRT inBore system in Ethos/Halcyon linear accelerator (Linac) systems for surface‐guided radiotherapy (SGRT), including developing a soft collision quality assurance (QA) to pass daily AlignRT QA without daily calibration, ensuring compatibility between SGRT and image‐guided radiotherapy (IGRT) setups, establishing a baseline performance in AlignRT motion monitoring, and enabling breast DIBH SGRT using manual beam gating with timing accuracy assessment.

**Methods:**

The AlignRT inBore system contains three ceiling‐mounted camera pods for SGRT setup at the simulation isocenter (sim‐ISO), and two ring‐mounted camera sets on the spring‐supported bore cover for motion monitoring at treatment ISO (txt‐ISO), after iterative cone‐beam computed tomography (iCBCT) setup. The commissioning includes confirming the ring cameras are outside the radiation fields, verifying IEC coordinate consistency between SGRT and IGRT, and ensuring the compatibility of AlignRT daily QA with collision QA. A dot‐array plate was used for SGRT calibration and daily QA at both ISO positions. A soft collision check method was developed to minimize the bore‐cover displacement by (1) triggering the collision alarm from the rear side, distant from the front ring cameras, and (2) reproducing the rear bore‐cover position by marking before the collision check. Clinical staff were trained for the new daily QA procedure, and the average period between calibrations was calculated over the past 12 months. Additionally, the application‐specific breast DIBH SGRT procedure was commissioned through an end‐to‐end test with manual gating, which was retrospectively evaluated for timing accuracy.

**Results:**

The AlignRT inBore camera ring was moved 2 cm away from the iCBCT field toward the bore front, and the IEC 61217 coordinate system yields consistent SGRT and IGRT setups. AlignRT inBore daily QA can pass using the soft collision procedure without daily calibration, with an average calibration period of 2 weeks, as ad‐hoc calibrations are required after open‐bore services. The manual gating accuracy is found to be 0.6s ± 0.2s, which can be further reduced if the beam can be proactively turned off before coaching patients to relax from a DIBH.

**Conclusion:**

The commissioning of the AlignRT inBore systems was successfully implemented, including developing and implementing the soft collision QA without daily calibration for 2 weeks, updating inBore camera position (2 cm to the bore front), ensuring SGRT‐IGRT couch‐shift consistency, establishing a baseline performance (< 0.2 mm drift), and allowing SGRT applications on Ethos/Halcyon systems. The application‐specific breast DIBH SGRT procedure with manual beam gating is also commissioned with a new retrospective timing analysis for assessing gating accuracy (0.6s ± 0.2s).

## 1 INTRODUCTION

For bore‐based linear accelerators (Linacs), such as Ethos and Halcyon systems (Varian, Palo Alto, CA), four commercial optical surface imaging (OSI) systems are available, including the AlignRT inBore system (VisionRT, London, UK), the IDENTIFY system (Varian, Palo Alto, CA), the Catalyst+ HD (C‐RAD, Uppsala, Sweden), and the LUNA 3D (LAP Laser, Luneberg, Germany). Currently, the Ethos/Halcyon systems do not have the motion management interface (MMI) to communicate with any of these optical surface imaging (OSI) systems, so the beam control cannot be performed automatically, but manually.^1^, ^2^, ^3^ The AlignRT inBore system was the first surface‐guided radiotherapy (SGRT) system available for the bore‐based Linacs, with a system configuration differing from that of the other vendors^4^, ^5^: it contains three ceiling‐mounted (external) camera pods aiming at the simulation isocenter (sim‐ISO) outside the bore for setup, and a bore‐mounted (internal) ring with two camera sets aiming at the treatment ISO (txt‐ISO) for motion monitoring. The external camera system is essentially the same as the AlignRT Advance system for the isocentric Linacs, such as TrueBeam systems. In contrast, the internal system is a new component, which ensures unobstructed visualization of the patient's region of interest (ROI) for motion monitoring during treatment. The field of view (FOV) for the internal cameras is slightly smaller than that of the external cameras, as the inBore cameras are close to the patient. Furthermore, the camera ring is mounted on a spring‐supported bore cover, and thus, the cameras are subject to movement, especially after the daily collision check during morning quality assurance (QA) as well as after open‐bore services. Clinically, although SGRT has been applied to treat left‐sided breast DIBH patients with manual gating,^6^ the latency of the manual gating procedure needs to be checked, as automatic gating is unavailable on the AlignRT inBore‐Ethos/Halcyon systems, unlike the AlignRT Advance‐TrueBeam with automatic gating.^7^, ^8^

A commissioning procedure of the AlignRT inBore system on Ethos/Halcyon was previously reported,^1^, ^2^ but a few critical issues remain to be addressed, including the IEC coordinate system consistency between SGRT and IGRT, the daily QA compatibility between AlignRT inBore and collision check, and end‐to‐end test with manual beam gating and timing evaluation. Three AlignRT inBore systems (on two Ethos and a Halcyon) were commissioned. Under the New York State regulations and the AAPM Task Group 142 guidelines,^9^ a daily collision check is required for any bore‐based Linacs to ensure patient safety. To trigger the collision alarm, the bore cover must be pushed to move about 2 cm, which may cause 2–3 mm residual shift of the bore cover, sufficient to invalidate the monthly calibration. Therefore, it became impossible to pass AlignRT inBore daily QA after the daily collision check without daily calibration,^1^ which not only increases physics workload but also the chances of human error.^4^, ^5^ Clinically, it became a practical barrier to applying SGRT on Ethos/Halcyon Linacs without skipping the daily collision check. In this technical note, we focus on three commissioning components of the AlignRT inBore system, complementary to the previous commissioning report.^1^, ^2^, ^5^ We reported the development and implementation of a soft collision check procedure that allows reproducing the bore cover position, so that daily inBore QA can pass (within the tolerance of 1.0 mm for the external cameras and 2.0 mm for the internal cameras) without calibration, overcoming the daily calibration requirement reported previously.^1^, ^2^ We also evaluated the SGRT stability and compatibility with IGRT. Additionally, we commissioned the manual gating procedure for left‐sided breast DIBH treatments. However, the SGRT for intracranial stereotactic radiosurgery (SRS) was not commissioned on Ethos and Halcyon because no non‐coplanar beams are available.^7^, ^10^

## 2 MATERIALS AND METHODS

**FIGURE 1:**
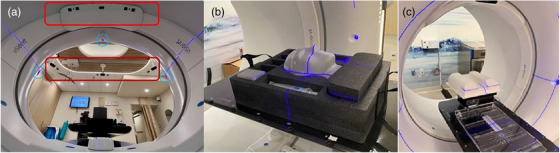
(a) The AlignRT inBore system with external (dark red box) and internal (bright red box) camera sets from a bore's eye view. The purple laser lines are shown aiming at the setup isocenter (sim‐ISO). (b) The experimental setup with the Steev head phantom for the SGRT‐IGRT consistency test and the OSI stability tests. (c) The experimental setting for the deep‐inspiration breath‐hold (DIBH) gating test using a QUAZAR mobile platform and a leg phantom.

The AlignRT inBore system, with its unique configuration, provides a sufficient FOV in bore‐based Linacs (Ethos or Halcyon) for real‐time surface guidance in patient SGRT setup and motion monitoring. In conjunction with IGRT, we conducted SGRT‐IGRT compatibility tests. In addition to AlignRT inBore system commissioning, we performed the procedure‐specific commissioning for breast DIBH with manual beam gating, including beam delivery consistency, end‐to‐end test, and sub‐second beam gating analysis. The AlignRT inBore system, the experimental setup using a head phantom (STEEV™, Sun Nuclear), and the QUAZAR mobile platform supporting a leg phantom for the commissioning are shown in Figure 1. Three AlignRT inBore systems on two Ethos and one Halcyon Linac were commissioned.

### 2.1 Consistency between SGRT and IGRT for patient setup

In the Ethos or Halcyon systems (v.4.0), iterative CBCT (iCBCT) using Varian's HyperSight imaging technology for fast scan with highly accurate Hounsfield Unit (HU) to provide planning quality CBCT for all treatment setups. The two Ethos systems were on a newly released version (v.4.0), so the compatibility between SGRT (AlignRT inBore) and IGRT (iCBCT) needs to be assessed during AlignRT inBore commissioning, as two different vendors are involved. First, the positioning of the inBore camera ring relative to the kV and MV beams can be assessed using the projection image to see if the ring camera is shown. In fact, we found the ring camera in the projection images by accident when performing kV profile correction in continuous dosimetry mode, so we decided to move the ring forward to the front by 2 cm after consulting with the SGRT vendor.

Second, the IEC coordinate system between SGRT and IGRT must be compatible, so that any positioning shifts detected by either SGRT or IGRT are consistent, ensuring correct patient setups. For both SGRT and IGRT experimental testing, the Steev phantom was immobilized on the couch with three degrees of freedom (3DOFs), and the sim‐ISO was set up to the middle of the brain using the Ethos’ laser system, as shown in Figure 1b. The SGRT and IGRT reference images were acquired, and the couch position was recorded based on the readings at the control panel.

### 2.2 Compatibility between daily inBore QA and bore collision QA

Under the New York State regulations and AAPM Task Group 142 guidelines,^9^ patient safety QA must be performed on a daily basis before treatment, including a collision check in a bore‐based Linac system. Four collision sensors are located about 2 cm behind the bore cover, which is suspended in space, supported by two spring‐loaded hangers behind the cover. When one of the sensors is touched, it triggers the collision interlock, freezing the Linac system's motion and terminating radiation. As the inBore camera ring is mounted on the bore cover, the collision check will alter the ring camera position by at least 2–3 mm, large enough to invalidate the monthly calibration. The failure in daily AlignRT QA after collision check was experienced, confirming the previous report that daily calibration was required during the morning QA.^1^

To find a solution to pass daily QA without daily calibration, we collaborated with the vendor to further immobilize the cameras in the ring, as well as develop a soft collision check procedure that enabled us to reproduce the bore cover position after a collision check. As the camera ring is mounted in the front of the bore, aiming at the txt‐ISO, we decided to trigger the collision from the rear end of the bore. Additionally, the white bore cover and grey machine cover are almost abutting to each other, so their relative position can be marked visually or physically before and reproduced after the collision check. Therefore, the motion of the ring cameras should be minimized, improving the chance of passing AlignRT inBore daily QA. Initial testing of inBore daily QA after daily soft collision check lasted more than a month, and the average time between calibrations was calculated based on the number of AlignRT calibrations in the past year, demonstrating the usefulness of the soft collision check.

### 2.3 The baseline‐drift uncertainties for both external and internal camera systems

The baseline‐drift errors of both external and internal cameras were measured using the same head phantom by applying real‐time‐delta (RTD) imaging for 20 min continuously. If the systems were affected by thermal heating from the projection lights, the RTD curves in 6DOF would gradually drift away from their all‐zero starting point and level off at a steady stage when a thermal equilibrium was reached. The leveling‐off values were the uncertainties, shown in the High‐Definition (HD) cameras with a light‐emitting device (LED) for sparkle light projection.^7^ For the AlignRT inBore systems, the latest Horizon cameras were used, which are supposed to be more thermally stable. Within 20 min before this experiment, RTD was not used on the external and internal cameras in the AlignRT inBore system, ensuring the cameras were “cold” (no continuous, excessive heat from the projector) at the beginning of the test. The heat‐induced camera baseline drift can be recovered after 15 min of idling without using RTD.^7^

### 2.4 SGRT for breast DIBH setup and monitoring with manual beam control

This is a procedure‐specific commissioning for the clinical breast DIBH workflow, including SGRT setup, DIBH motion monitoring, cone‐beam CT (CBCT) verification, and manual beam control and gating timing assessment. Machine output consistency for Ethos and Halcyon Linacs was tested, comparing gated and non‐gated beam delivery using an A12 ion chamber inside a solid water phantom placed at the txt‐ISO. A 3‐arc breast VMAT plan (520 cGy) was applied, and two manual beam holds were used: (1) Press and hold the BeamOn button to hold and release it to resume the beam, and (2) Press the BeamOff button to stop the beam and manually restart the beam to resume. The manual beam on/off to deliver the VMAT plan was conducted by a therapist. A typical patient's DIBH motion trace was loaded into a QUAZAR mobile platform to create repeated vertical motion of the leg phantom (Figure 1c), which was monitored by RTD of the AlignRT inBore ring camera set. The reference DIBH image captured at sim‐ISO outside the bore was used at txt‐ISO by switching the external to the internal camera set.

The RTD data were retrieved from the AlignRT server system, while the beam on/off data were retrieved from the Ethos or Halcyon systems’ log files. The Varian treatment workstation clock was synchronized to the Juniper NTP timer biweekly, while AlignRT systems were synchronized to an internal MSKCC timer daily. The time difference was verified to be about 100 ms (within 300 ms) using the NISTimer (National Institute of Standards and Technology). Using both log files, the DIBH manual gating timing accuracy was assessed.

## 3 RESULTS

### 3.1 Consistency between SGRT and IGRT for setup

**FIGURE 2:**
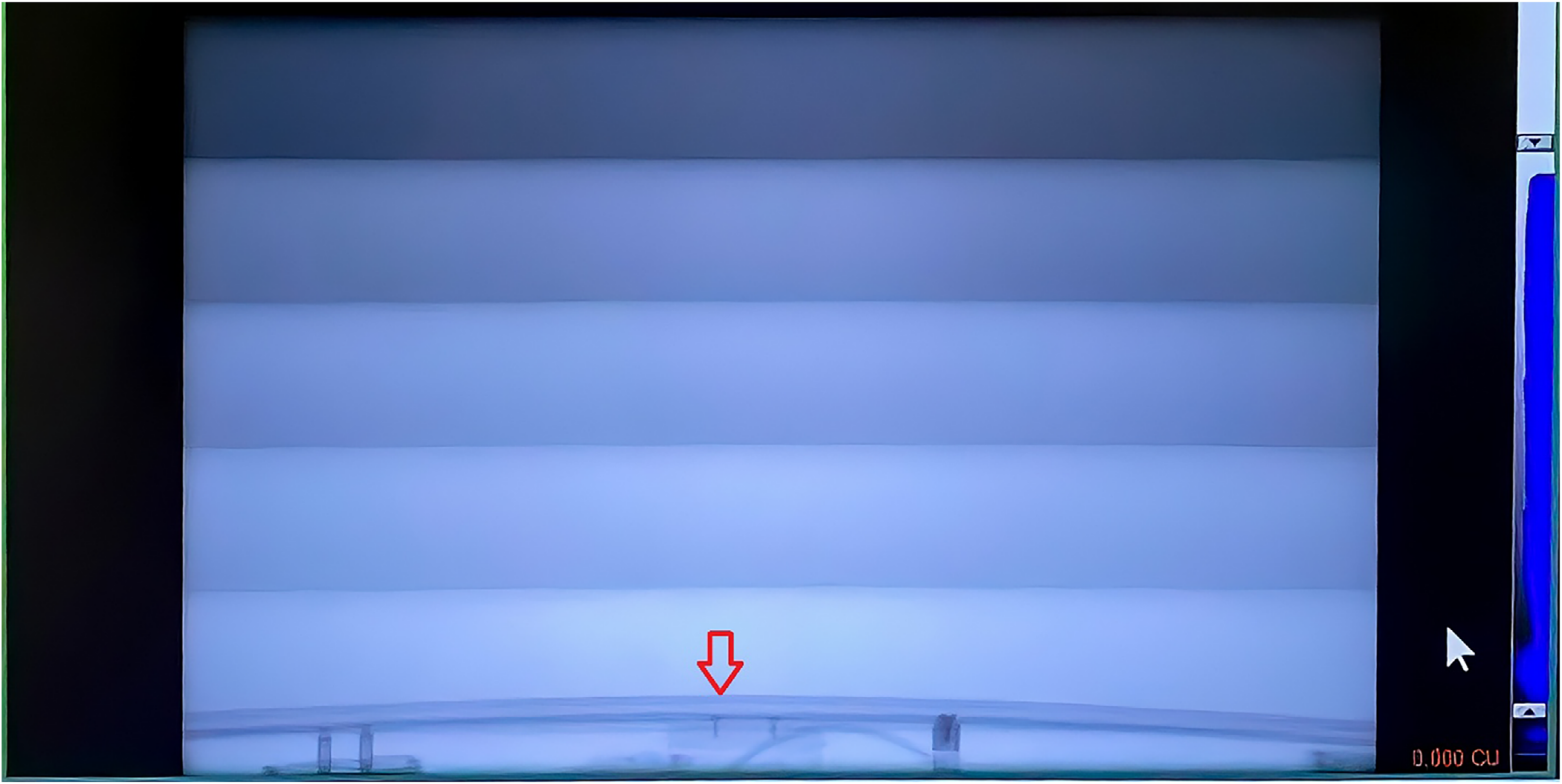
A radiographic image (without profile correction) shows that the inBore camera ring was 1.73 cm into the beam field (red arrow at the bottom) and was moved by 2 cm toward the front of the bore, because the bigger HyperSight detector was introduced, while the bore cover was unchanged.

Figure 2 is a radiographic projection image acquired for beam profile correction, showing the bore‐mounted camera ring inside the beam field as a bigger HyperSight detector was introduced in Ethos v4.0. This finding led us to move the camera ring forward by 2 cm, outside the kV beam field, ensuring iCBCT image quality and avoiding unnecessary radiation to the ring cameras. In the recent SGRT commissioning on Halcyon, the ring camera mounting was updated: no shift is necessary.

Table 1 tabulates the results of detected phantom shifts from iCBCT and AlignRT inBore systems, which were set in the IEC 61217 coordinate systems. The detected couch shifts are compared, and the residual shifts in AlignRT inBore are shown after iCBCT setup correction.

**TABLE 1:** The consistency of SGRT (AlignRT inBore) and IGRT (iCBCT) under the IEC 61217 coordinate system for both systems. An arbitrary translational shift (0.40, −0.40, −0.40) (in cm) was set and applied to the phantom from the reference position, and the detected, corrected, and residual shifts by both systems were shown. The measured actual shifts indicated by SGRT and IGRT are tabulated in this table.

		AlignRT (cm)			CBCT (cm)			Couch (cm)		
Test	Condition	VRT	LNG	LAT	VRT	LNG	LAT	VRT	LNG	LAT
Setup	sim‐ISO	0.01	−0.01	0.00				−23.4	45.6	1.4
	txt‐ISO	0.04	−0.05	−0.02	0.07	−0.06	−0.10	−9.3	92.9	0.2
	CBCT Setup	−0.02	0.01	0.07	0.02	0.00	0.00	−9.2	92.8	0.1
Shifts	Introducing couch shifts (−0.40, 0.40, 0.40) (cm)									
	Initial	0.36	−0.37	−0.35	0.40	−0.39	−0.41	−9.6	93.2	0.5
	CBCT Corrected	−0.04	0.01	0.07				−9.2	92.8	0.1

### 3.2 Compatibility between AlignRT inBore QA and collision check

**FIGURE 3:**
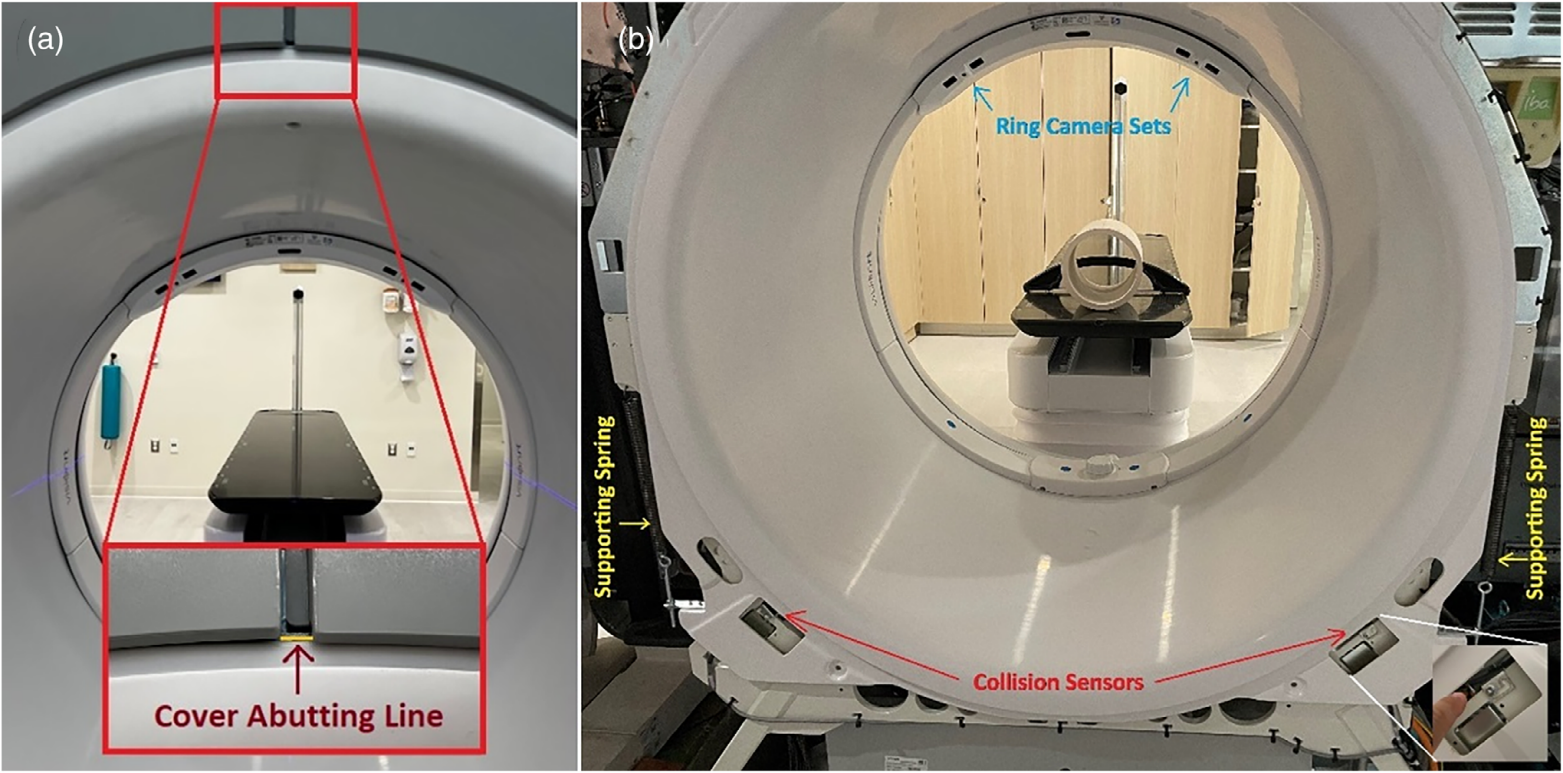
Rear views of the abutting position between the bore cover (white) and machine cover (grey) (a), and two supporting springs, and two collision sensors (enlarged in the insert) when the machine cover is open (b). The front‐located, bore‐mounted ring camera sets are shown.

**FIGURE 4:**
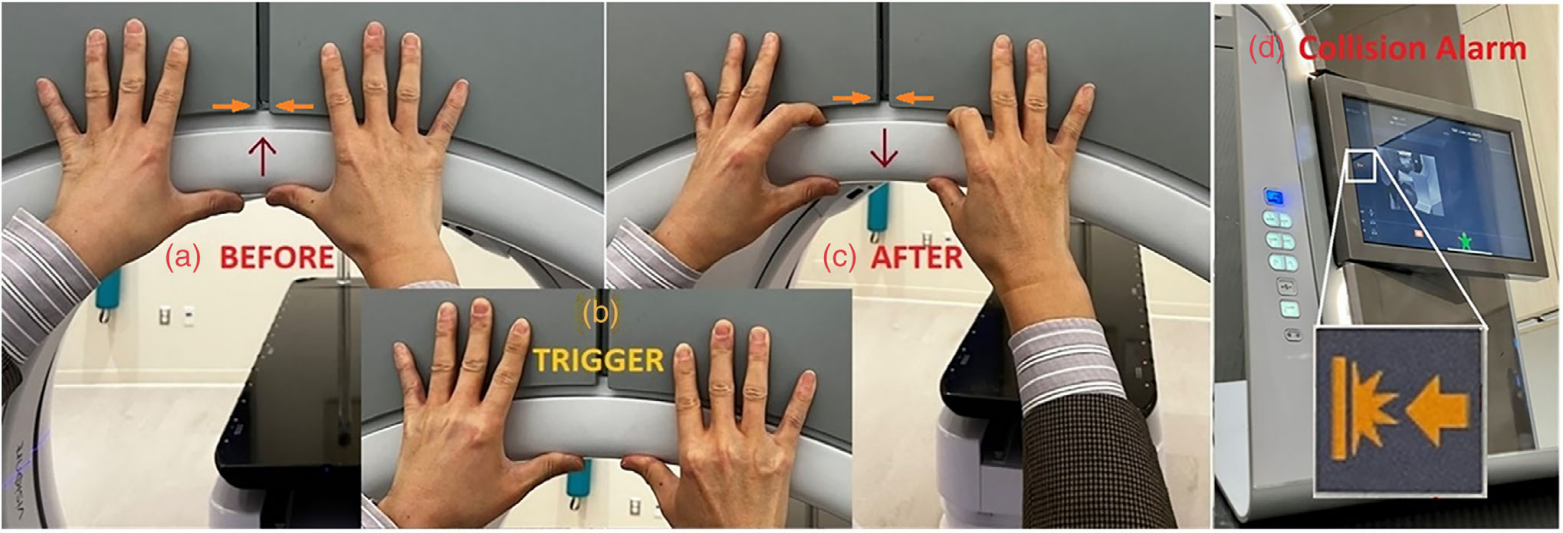
Illustration of the soft collision check process: (a) Marking the initial abutting edge position (orange arrows) and pushing the bore cover upward to trigger a collision with both thumbs, (b) relaxing the thumbs to allow gravity to pull down the cover, (c) using both index fingers to tap the bore cover down to reach its initial, marked position (orange arrows), and (d) the collision error sign in the front panel can be viewed via a mirror placed on the couch (the insert shows the orange blinking collision alarm sign), or by a second staff.

**FIGURE 5:**
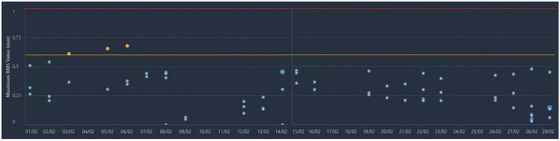
Demonstration of successful daily QA of the AlignRT inBore system after the soft daily collision check for 4 weeks without recalibration. The yellow line (at 0.6 mm) provides a warning (yellow data points), and the red line (at 1.0 mm) indicates a failure of daily QA.

Figure 3a shows the rear view of the Ethos bore cover (white), machine cover (grey), and their abutting position (the yellow line in the insert). Figure 3b shows the two supporting springs and two rear collision sensors when the machine cover is opened. The bore‐mounted camera ring is also shown at the front of the inner bore cover. Figure 4 illustrates the soft collision triggering workflow from the rear end by marking the two‐cover abutting position, using fingers to trigger the collision, and reproducing the abutting bore cover position after the collision check. Figure 5 demonstrates a 4‐week continuous daily QA record without the need for AlignRT inBore recalibration. Based on the calibration record in the past 12 months, the calibration of the AlignRT inBore system occurred every 2 weeks (13.9 ± 9.6 days) on average, ranging from 3 to 31 days. Among a total of 26 calibrations, 7 were performed before 8:00 am, likely caused by daily QA failure, while 19 were regular monthly or ad hoc calibrations. It is worthwhile to note that an ad hoc calibration must be performed after bore‐opening services.

A checklist of morning QA for the AlignRT inBore system with an Ethos/Halcyon system is provided below:

1. Perform and complete the Machine Performance Check (MPC), and any necessary clinic‐required, procedure‐specific morning QA.
2. Place a mirror on the couch to view the front control panel from the rear of the bore (Figure 4) or ask another staff member to watch the control panel.
3. Perform the soft collision check from the rear end of the bore to trigger the collision alarm and reset the bore cover (Figures 3 and 4).
4. Perform AlignRT inBore daily QA, which will pass most times. In case daily QA fails, call the physicist to redo the AlignRT inBore system calibration.
5. Retrieve the AlignRT daily QA and calibration history record and graphic display (Figure 5) using the Report feature for retrospective analysis.

### 3.3 Stability for RTD motion monitoring for setup and treatment

**FIGURE 6:**
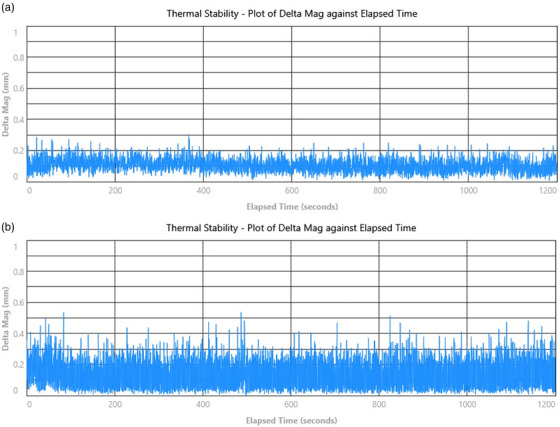
The thermal stability in 20 min for both external (a) and internal (b) cameras.

Figure 6 demonstrates the thermal stability of the external and internal cameras over 20 min. No apparent baseline drift and only background noise at 0.2–0.3 mm is observed. This finding is consistent with a previous report on the Horizon cameras,^2^ but smaller in magnitude than previous HD cameras.^7^

### 3.4 Commissioning of breast DIBH procedure with manual beam control

**FIGURE 7:**
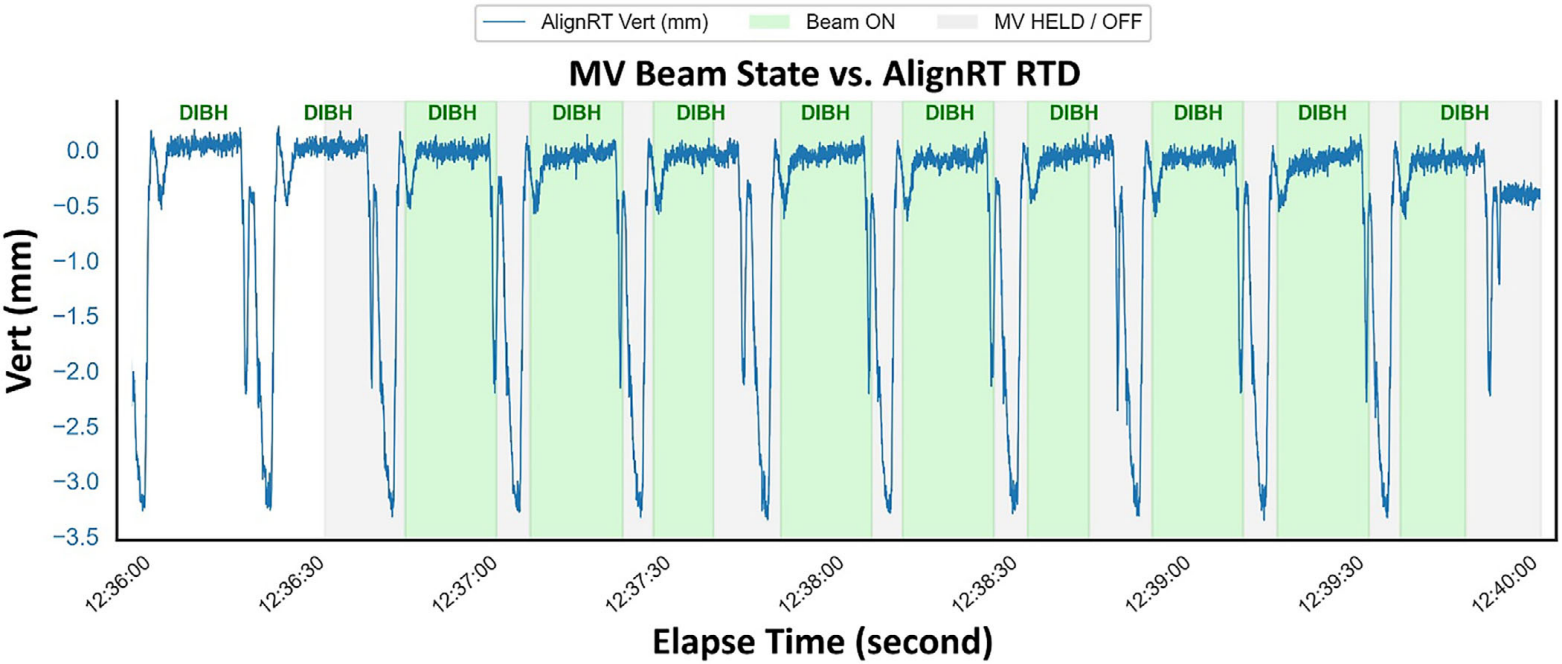
Beam On/Off status from Varian's log file versus AlignRT real‐time delta (RTD) file for DIBH vertical motion monitoring using a testing DIBH curve on the Halcyon system. In three out of the nine DIBH replicating curves, the beam finished earlier than the DIBH, and they were excluded from the analysis (Table 2).

Table 2 tabulates dosimetry tests and manual gating timing tests (see Figure 1c: the setting for the DIBH gating test), and Figure 7 shows the gating timing analysis by comparing the AlignRT RTD file (DIBH motion curve) and the Ethos log file (beam control events).

**TABLE 2:** Dosimetry error for gated delivery in reference to non‐gated delivery, and timing error of manual beam control by comparing synchronized.

Linac	Gating Error vs. No Gating (MU and %MU)			Manual DIBH Beam Off Timing Error (s)		
	Average	St Dev	Relative Error (%)	DIBH #	Average	St Dev
Ethos 1	2.0	2.4	−0.1	9	0.60	0.22
Ethos 2	2.3	3.6	0.1	‐	–	‐
Halcyon	2.4	1.9	0.1	6	0.61	0.04

## 4 DISCUSSION

### 4.1 Ensuring the compatibility between SGRT and IGRT

Unlike the AlignRT Advance system, the AlignRT inBore system has a camera ring that is mounted on the inner bore of the Ethos/Halcyon systems, and therefore, its compatibility with the host system must be checked during the commissioning process. The Ethos system (v.4.0) had upgraded its kV detector to the HyperSight imaging detector, bigger than the previously used conventional kV detector, although the bore cover remained the same, so that the conventional inBore ring mounting position was found inside the projection image (Figure 2). After consulting with the vendor, we moved the inBore camera ring by 2 cm toward the front end. This is necessary to ensure CBCT quality for patient setup, as well as for online planning for adaptive treatments. In the recent commissioning of the AlignRT inBore system on a Halcyon Linac, the change in the ring camera position has been incorporated.

Additionally, the SGRT‐IGRT consistency under the IEC 61217 coordinate system for both AlignRT inBore and Ethos systems was verified, so that the shifts from SGRT and IGRT setups are consistent, as shown in Table 1. This IEC coordinate system for the AlignRT inBore system on Ethos/Halcyon system is different from that of the AlignRT Advance system on TrueBeam.^7^ Such a comparison is necessary to ensure a consistent patient setup between SGRT and IGRT.

### 4.2 Enabling SGRT on bore‐based Linac compliant with AAPM TG‐142

Because a daily collision check moves the bore cover beyond the tolerance of daily AlignRT inBore QA, it requires daily calibration in order to pass the daily QA, so the system can be used for clinical SGRT.^1^ Performing daily calibration is impractical as it requires a physicist to be available during the morning QA, which adds workload for physicists and increases the chance of human error. So, daily collision checks are often omitted in some clinics, despite AAPM Task Group 142 guidelines. Here, the establishment of the soft collision check allows inBore daily QA to pass, so daily calibration is no longer needed, making SGRT practical in the clinics under the New York State regulation. Since February 2024, the soft collision check procedure has been found reliable and consistent to pass daily AlignRT inBore QA without calling physicists for calibration on our two AlignRT‐inBore‐Ethos systems.

Additionally, although breast DIBH with manual beam gating on Ethos/Halcyon has been reported for patient treatment,^6^ the procedure‐specified commissioning has not been reported. This procedure‐specific commissioning provides validation and verification methods for manual gating accuracy. This timing analysis can also be applied for a retrospective investigation in case of clinical complications.

### 4.3 Opportunities to develop more SGRT procedures on Ethos/Halcyon systems

As the iCBCT imaging is required for any treatments, a pair of SGRT and IGRT datasets would be available for every treatment fraction during the course of radiotherapy. Therefore, it offers a unique opportunity to conduct prospective studies to compare the daily OSI with the daily iCBCT, assessing SGRT setup accuracy. This is especially valuable when developing a new SGRT procedure for a new disease site, as tattooless and markerless SGRT approaches are progressively dominating patient setups, replacing conventional laser‐based setup techniques.^4^, ^5^, ^11^ For instance, we have employed SGRT for HN patient setup and treatment, using a two‐ROI strategy to control the neck (the cervical spine) curvature at setup.^12^ The direct comparison of the patients’ SGRT and IGRT setups, together with the deep‐learning‐based auto‐segmented internal organs,^13^ allows us to study and apply SGRT to infer internal structure alignment.^14^ We have applied the SGRT on other disease sites, including head and neck, breast, bladder, and prostate. The unique datasets are valuable for further investigation of respiratory motion management using deep‐learning‐based modeling approaches.^15^, ^16^

### 4.4 Limitations of this commissioning report

This should serve as a complementary supplement to previous commissioning reports of the AlignRT inBore systems for Ethos/Halcyon systems,^1^, ^2^ since it introduces some new commissioning contents. For instance, the new daily QA procedure without the requirement for daily calibration, the SGRT‐IGRT consistency test, and procedure‐specific breast DIBH tests and gating timing analysis. However, we did not perform intracranial SRS commissioning as the Ethos/Halcyon system does not provide non‐coplanar beams, which are necessary for optimal SRS plans and delivery.^7^ This report only covers the AlignRT inBore system, and some of the details may not apply to other commercial OSI systems for SGRT applications.^4^, ^5^ For instance, the soft collision check procedure is only tested on Varian's Ethos and Halcyon systems, and the manual gating accuracy can only be checked manually and retrospectively, although it is possible to implement an automatic timing assessment in the future. Additionally, these new procedures require sufficient clinical training for therapists to use them properly daily.

## 5 CONCLUSION

This report on the commissioning of the AlignRT inBore system for Ethos/Halcyon systems provides additional information complementary to previous reports providing solutions for clinical SGRT applications, including ensuring collision‐inBore QA compatibility by developing a soft collision QA to eliminate the need for daily calibration for two weeks on average, updating inBore camera position compatible with the HyperSight™ imaging detector, verifying SGRT‐IGRT couch‐shift consistency, and establishing a baseline of the system performance. Additionally, application‐specific breast DIBH SGRT procedure with manual beam gating is commissioned with a new retrospective timing analysis to ensure sub‐second gating accuracy.

## AUTHOR CONTRIBUTIONS

Guang Li: Designed and developed the soft collision check procedure, participated in the AlignRT inBore‐Ethos commissioning, and wrote and revised the article, including the point‐by‐point responses in the review process. Xiaoning Liu: Major contribution to conducting the AlignRT inBore commissioning. William Donahue: Contribution to the commissioning of the Ethos aspect, collision checks. Shih‐Chi Lin: Discovered the AlignRT inBore camera ring was inside the Ethos kV beam field. Chengzhu Zhang: Conducted the breast DIBH SGRT commissioning for the Halcyon system. Wendy Harris: Conducted the breast DIBH SGRT commissioning for the Halcyon system. David Kanchaveli: Contribution to testing the inBore daily QA and calibration. Seng Boh Lim: Followed NYS regulations and AAPM guidelines for Ethos‐AlignRT‐inBore for commissioning and SGRT applications. Michell Savacool: Assisted with testing AlignRT QA and collision QA. Michalis Aristophanous: Discussion on the AlignRT inBore system commissioning for SGRT. Laura Cervino: Discussion on the AlignRT inBore system commissioning for SGRT.

## CONFLICT OF INTEREST STATEMENT

The authors declare no conflicts of interest

## Data Availability

If needed, we will make efforts to make the data used in this study available upon the readers’ request.
